# Effects of rivaroxaban combined with clopidogrel on thrombotic risk and myocardial injury indicators in patients with coronary artery ectasia

**DOI:** 10.3389/fcvm.2026.1912975

**Published:** 2026-10-09

**Authors:** Tao Geng, Mengwei Feng, Yunjie Wu, Zhaoqing Xie, Jingxian Xing, Deguang Wang, Xue Liang, Juan Liu, Hui Gao

**Affiliations:** 1 Department of Cardiology, Cangzhou Central Hospital of Hebei Medical University, Cangzhou City, Hebei Province, China; 2 Department of Cardiology, The Second Hospital of Tianjin Medical University, Tianjin, China

**Keywords:** antithrombotic therapy, clopidogrel, coronary artery ectasia, myocardial injury, platelet activation, rivaroxaban, thrombotic risk

## Abstract

**Objective:**

To investigate the association between rivaroxaban combined with clopidogrel therapy and thrombotic risk, platelet activation, myocardial injury biomarkers, and clinical outcomes in patients with coronary artery ectasia (CAE).

**Methods:**

This single-center retrospective cohort study included 158 consecutive patients with CAE diagnosed by coronary angiography between June 2023 and June 2025. Patients receiving clopidogrel alone or rivaroxaban plus clopidogrel were compared. A 1:1 nearest-neighbor propensity score matching method was applied, generating 70 matched pairs. Thrombotic risk markers, including D-dimer (D-D), thrombin-antithrombin complex (TAT), and von Willebrand factor (vWF), platelet activation parameters [ADP-induced platelet aggregation rate (ADP-PAg) and CD62P], thromboelastography parameters [maximum amplitude (MA) and reaction time (R)], and myocardial injury-related biomarkers [high-sensitivity cardiac troponin T (hs-cTnT), creatine kinase-MB (CK-MB), heart-type fatty acid-binding protein (H-FABP), and N-terminal pro-B-type natriuretic peptide (NT-proBNP)] were evaluated at baseline and 6 months. Clinical outcomes, including thrombotic-related adverse cardiovascular events and bleeding events, were recorded during 12 months of follow-up.

**Results:**

After propensity score matching, baseline characteristics were well balanced between groups. At 6 months, patients receiving rivaroxaban plus clopidogrel showed lower levels of D-D, TAT, vWF, ADP-PAg, CD62P, MA, hs-cTnT, CK-MB, H-FABP, and NT-proBNP, as well as prolonged R values, compared with those receiving clopidogrel alone (all *P* < 0.05). During follow-up, thrombotic-related adverse cardiovascular events occurred less frequently in the combination therapy group than in the clopidogrel group (7.14% vs. 20.00%, *P* = 0.027). Minor bleeding events were numerically higher in the combination therapy group (17.14% vs. 10.00%, *P* = 0.218), while no intracranial or fatal bleeding events occurred.

**Conclusion:**

Among patients with CAE, rivaroxaban combined with clopidogrel was associated with more favorable changes in coagulation activation, platelet activity, myocardial injury biomarkers, and thrombotic-related clinical outcomes compared with clopidogrel alone. These findings suggest that combined anticoagulant and antiplatelet therapy may represent a potential strategy for selected high-risk patients with CAE. Further multicenter prospective studies are required to confirm these observations.

## 1 Introduction

Coronary artery ectasia (CAE) is a special type of coronary artery disease, mainly characterized by localized or diffuse abnormal dilatation of the coronary artery, usually defined as a lesion in which the diameter of the affected vessel reaches 1.5 times or more that of the adjacent normal coronary segment (1). With the widespread application of coronary angiography and coronary computed tomography angiography, the detection rate of CAE in clinical practice has gradually increased. Previous studies (2, 3) have shown that CAE may exist alone or coexist with coronary atherosclerotic stenosis, acute coronary syndrome, or coronary slow flow. Although CAE appears as vascular dilatation on imaging, this does not necessarily indicate favorable blood perfusion, and the clinical management of these patients remains challenging. Some patients may remain asymptomatic for a long time, whereas others may develop chest pain, myocardial ischemia, or even acute coronary events due to thrombosis or distal microcirculatory dysfunction (4, 5).

The pathogenesis of CAE is complex and not fully elucidated. Accumulating evidence suggests that CAE represents an exaggerated form of positive vascular remodeling in response to atherosclerosis, driven by chronic inflammation, endothelial dysfunction, and destruction of elastic fibers in the vessel wall (6). Unlike obstructive coronary lesions, the dilated segments in CAE exhibit abnormal hemodynamics characterized by slow blood flow, vortex formation, and blood stasis. These flow disturbances, combined with a heightened inflammatory state within the ectatic vessel wall, create a prothrombotic milieu that predisposes patients to local thrombosis, distal microembolization, and recurrent myocardial ischemia, thereby increasing the risk of adverse cardiovascular events (7). Consequently, patients with CAE may experience angina, acute coronary syndrome, or even sudden cardiac death in the absence of significant coronary stenosis (8).

Currently, there is no unified antithrombotic treatment guideline specifically for patients with CAE (8). In clinical practice, treatment is mostly based on the principles of coronary atherosclerotic heart disease management, including antiplatelet therapy, lipid regulation, and comprehensive risk factor control. As a commonly used P2Y12 receptor antagonist, clopidogrel can effectively inhibit platelet activation and aggregation and is widely used in the secondary prevention of coronary heart disease (9). However, the mechanism of thrombosis in patients with CAE is not only related to platelet activation; slow blood flow within dilated vessels and activation of the coagulation system also play important roles. Antiplatelet therapy alone may not fully interfere with thrombin generation and fibrin formation. Therefore, whether further anticoagulant therapy should be combined with antiplatelet therapy in some patients with CAE at high thrombotic risk remains worthy of investigation.

Rivaroxaban is a direct oral factor Xa inhibitor that exerts an anticoagulant effect by inhibiting the coagulation cascade and reducing thrombin generation (10). In recent years, the concept of “dual-pathway antithrombotic therapy,” namely the combination of antiplatelet agents and low-dose anticoagulants, has attracted increasing attention. For patients with CAE, clopidogrel mainly acts on the platelet aggregation pathway, while rivaroxaban acts on the coagulation system; their combination may reduce thrombotic risk through different pathways. Based on this, the present study selected patients with CAE who were hospitalized in the Department of Cardiovascular Medicine of our hospital and confirmed by coronary angiography as the study subjects. A retrospective cohort study design was used to compare the clinical efficacy and safety of rivaroxaban combined with clopidogrel vs. clopidogrel monotherapy, with a focus on analyzing its effects on thrombotic risk and myocardial injury status, aiming to provide a clinical reference for the selection of individualized antithrombotic treatment strategies for patients with CAE.

## 2 Materials and methods

### 2.1 Study design and participants

This study adopted a single-center retrospective cohort study design. Patients who were hospitalized in the Department of Cardiovascular Medicine of our hospital from June 2023 to June 2025 and were definitively diagnosed with CAE by coronary angiography were consecutively selected as the study subjects. Clinical data were collected through the hospital electronic medical record system, coronary angiography database, laboratory information system, and follow-up record system. A total of 158 patients who met the diagnostic criteria for CAE were initially retrieved. After screening according to the inclusion and exclusion criteria, 18 patients were excluded, leaving 140 patients who entered the propensity score matching (PSM) procedure. Using 1:1 nearest-neighbor matching, 70 patients in the observation group were successfully matched with 70 patients in the control group. According to the different antithrombotic treatment regimens after discharge, the patients were divided into a control group (clopidogrel monotherapy, *n* = 70) and an observation group (rivaroxaban + clopidogrel, *n* = 70). Both groups received standardized secondary prevention for coronary heart disease and comprehensive management of risk factors. The study protocol was reviewed and approved by the Medical Ethics Committee of our hospital (Ethics Approval No.: 24XNLC-YWBJ05). Since this study was a retrospective analysis of clinical data, and all patient data were anonymized before collection and analysis without involving any additional diagnostic or therapeutic intervention, the ethics committee approved a waiver of written informed consent. The study was conducted in accordance with the relevant principles of the Declaration of Helsinki. The study flow diagram is shown in Figure 1.

**Figure 1:**
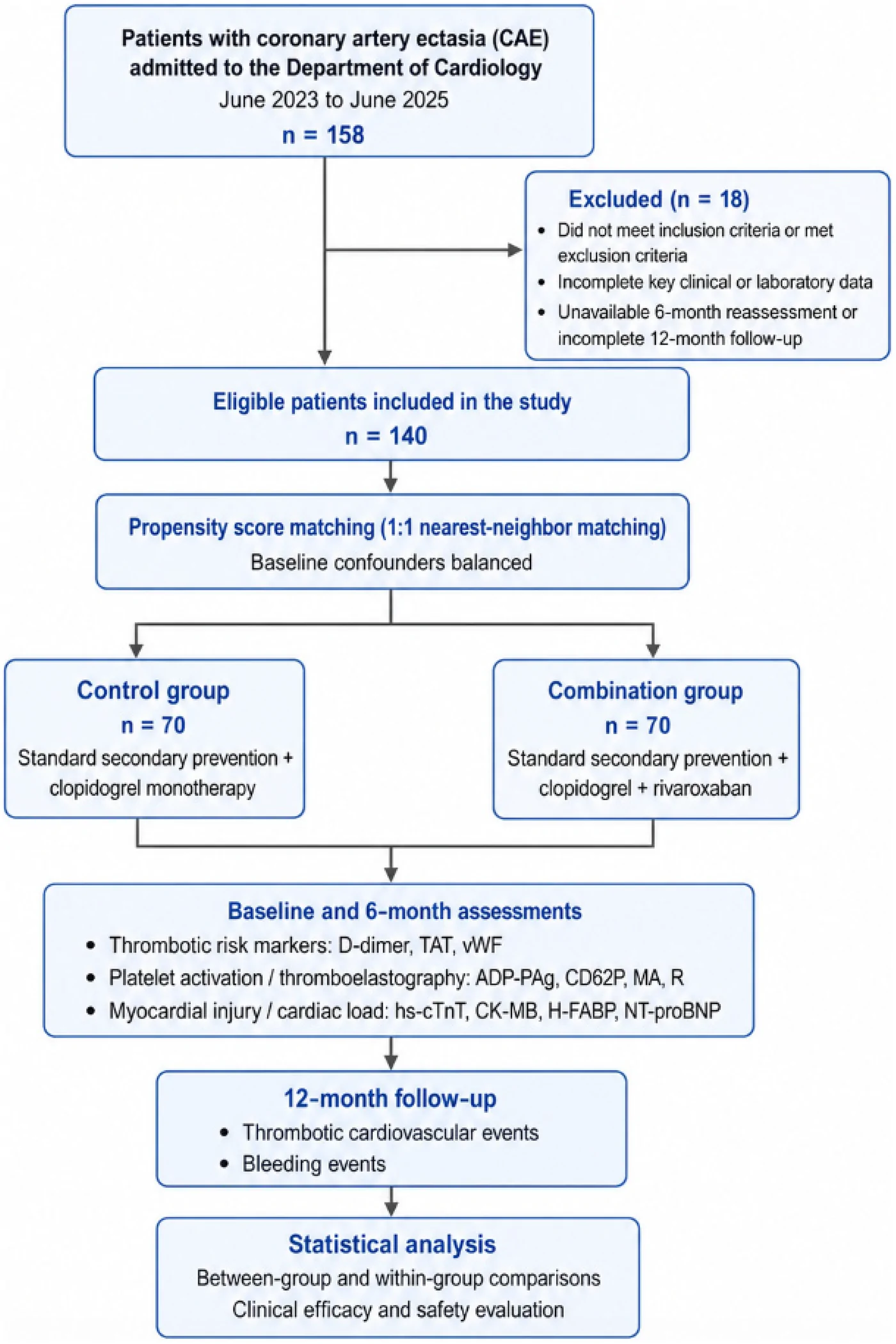
Flowchart of patient enrollment and study design.

### 2.2 Diagnostic criteria for CAE

The diagnosis of CAE was mainly based on coronary angiography findings. CAE was diagnosed when the luminal diameter of one or more segments of a coronary artery reached 1.5 times or more that of the adjacent normal coronary artery segment. Coronary angiography was performed by qualified cardiovascular interventional physicians, and the angiographic findings were independently interpreted by two experienced cardiovascular interventional physicians. If the interpretations of the two physicians were inconsistent, a third senior interventional physician reviewed the images to determine the final diagnosis. The involved coronary vessels, lesion extent, presence or absence of concomitant coronary artery stenosis, and degree of dilatation were recorded. The lesion extent of CAE was recorded according to the Markis classification (11) (the diagnostic criteria are shown in Figure 2). Type I was defined as diffuse ectasia of two or three coronary arteries; Type II was defined as diffuse ectasia of one coronary artery accompanied by localized ectasia of another coronary artery; Type III was defined as diffuse ectasia of a single coronary artery; and Type IV was defined as localized or segmental ectasia of a single coronary artery.

**Figure 2:**
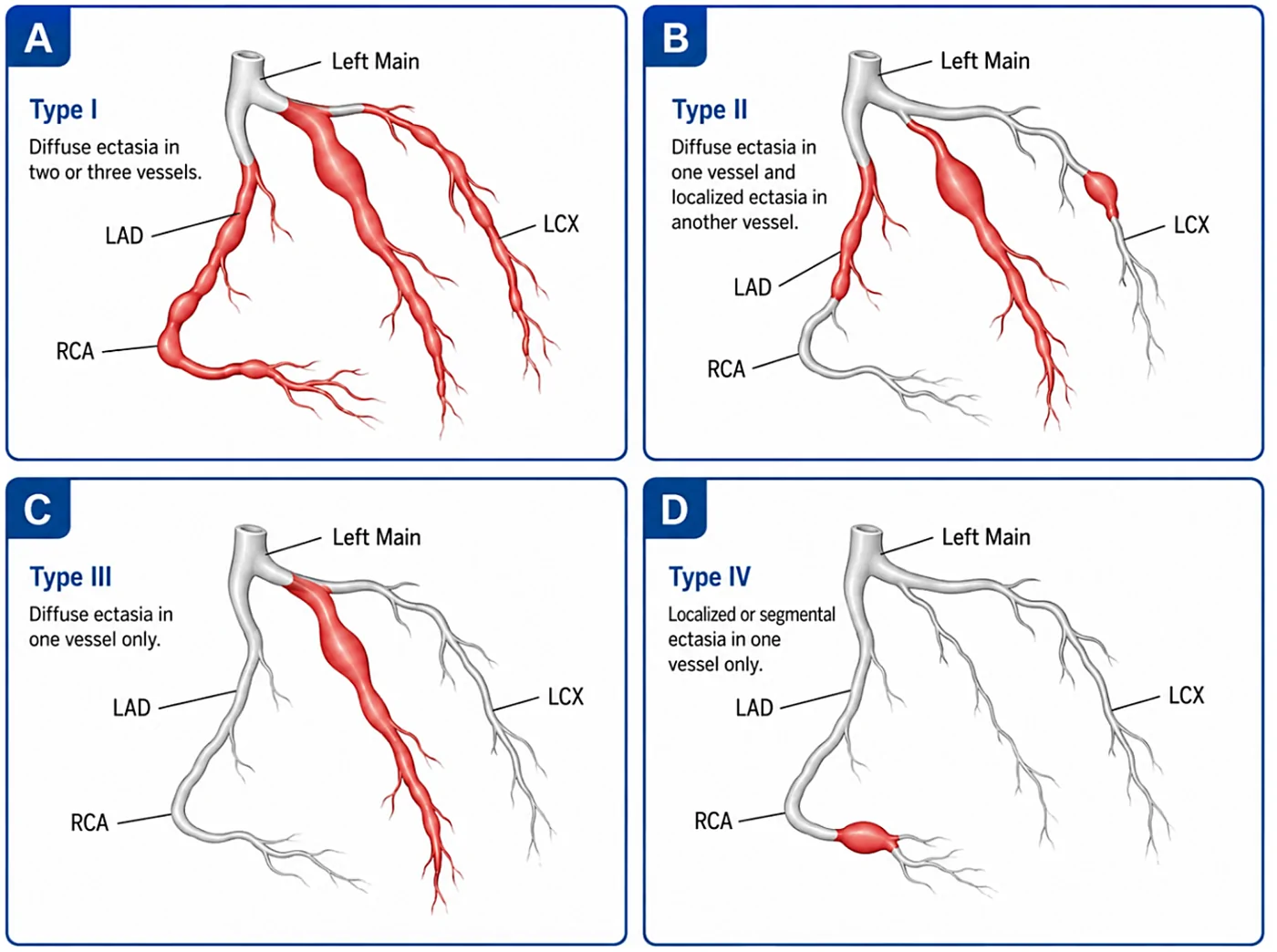
Schematic illustration of the markis classification of coronary artery ectasia **(A)** type I: diffuse ectasia involving two or three coronary arteries; **(B)** type II: diffuse ectasia of one coronary artery with localized ectasia of another; **(C)** type III: diffuse ectasia of a single coronary artery; **(D)** type IV: localized or segmental ectasia of a single coronary artery.

### 2.3 Inclusion and exclusion criteria

#### 2.3.1 Inclusion criteria

(1) Age ≥18 years, regardless of sex; (2) definite diagnosis of CAE by coronary angiography during hospitalization; (3) relatively complete clinical data, including general information, laboratory examinations, coronary angiography findings, treatment regimen, and follow-up data; (4) regular clopidogrel monotherapy after discharge, or rivaroxaban combined with clopidogrel treatment; (5) completion of relevant laboratory indicator re-examination at baseline and 6 months post-treatment; (6) completion of at least 12 months of clinical follow-up.

#### 2.3.2 Exclusion criteria

(1) Patients with definite indications for long-term anticoagulation, such as atrial fibrillation, mechanical valve replacement, or venous thromboembolism; (2) patients who experienced severe bleeding events within 3 months before admission, or those with a previous history of intracranial hemorrhage or active gastrointestinal bleeding; (3) patients with severe hepatic insufficiency, severe renal insufficiency, or coagulation dysfunction; (4) patients with markedly abnormal platelet counts or hematological diseases; (5) patients with malignant tumors, systemic immune-inflammatory diseases, severe infection, or recent major surgery; (6) patients with contraindications, allergy, or intolerance to clopidogrel or rivaroxaban; (7) pregnant or lactating women; (8) patients who discontinued medication, changed medication, or had poor compliance during treatment, thereby affecting efficacy evaluation; (9) patients with missing follow-up data or loss to follow-up.

### 2.4 Data collection

General clinical data, previous medical history, laboratory examination results, imaging data, medication information, and follow-up data were collected through the hospital electronic medical record system. General data included age, sex, body mass index (BMI), smoking history, drinking history, hypertension, diabetes mellitus, hyperlipidemia, previous history of coronary heart disease, previous history of percutaneous coronary intervention, and previous history of myocardial infarction. Clinical data included the main diagnosis at admission, clinical manifestations, blood pressure, heart rate, liver and kidney function, blood lipids, blood glucose, routine blood tests, and coagulation function. Coronary angiography data included the coronary arteries involved by CAE, lesion extent, presence or absence of concomitant stenotic lesions, degree of stenosis, whether percutaneous coronary intervention was performed, and Markis classification. Medication data included the type, dose, and treatment duration of antithrombotic drugs, lipid-lowering drugs, β-blockers, angiotensin-converting enzyme inhibitors or angiotensin II receptor blockers, calcium channel blockers, nitrates, and hypoglycemic drugs. To reduce data extraction bias, a unified data extraction form was developed before data collection. Two researchers independently extracted and entered the data. After data entry, cross-checking was performed. In case of inconsistencies, a third researcher reviewed the original medical records for verification. Missing data were recorded item by item. If key variables were missing and could not be supplemented, the patient was not included in the final analysis.

### 2.5 Propensity score matching method

Since this study was a retrospective cohort study and the treatment regimens were not randomly assigned, propensity score matching was used to reduce baseline differences and the influence of potential confounding factors on the study results. Whether the patient received rivaroxaban combined with clopidogrel treatment was used as the dependent variable. Variables that might affect the selection of antithrombotic treatment regimens and prognosis were included in the propensity score model, including age, sex, BMI, smoking history, hypertension, diabetes mellitus, hyperlipidemia, previous history of coronary heart disease, previous history of myocardial infarction, CAE lesion extent, Markis classification, presence or absence of concomitant coronary artery stenosis, and baseline medication treatment. A 1:1 nearest-neighbor matching without replacement was performed using a caliper width of 0.05 of the propensity score. After matching, 70 patients were successfully matched in the control group and 70 patients in the observation group. Baseline data between the two groups were compared after matching. If there were no statistically significant differences in major covariates between the groups and the standardized differences were markedly reduced, the two groups were considered to have good comparability after matching In this study, “baseline” was defined as the time point of hospital admission when the patient underwent diagnostic coronary angiography and the initial laboratory tests were performed. All baseline clinical data, laboratory parameters, and angiographic findings were collected at or before this time point. The 6-month post-treatment assessment was conducted approximately 6 months after discharge, corresponding to 6 months after the initiation of the assigned antithrombotic regimen.

### 2.6 Treatment methods

#### 2.6.1 Basic treatment

After admission, both groups received standardized comprehensive treatment according to the guidelines for the management of secondary prevention of coronary atherosclerotic heart disease (12) and the specific conditions of each patient. Basic treatment mainly included: (1) lipid-lowering therapy: patients without contraindications were treated with statins, such as atorvastatin or rosuvastatin, and ezetimibe was added when necessary to control low-density lipoprotein cholesterol levels; (2) anti-myocardial ischemia therapy: β-blockers, calcium channel blockers, or nitrates were used according to the patients' symptoms, heart rate, and blood pressure; (3) blood pressure and blood glucose management: individualized antihypertensive and hypoglycemic treatments were given to patients with hypertension or diabetes mellitus; (4) risk factor control: patients were instructed to quit smoking, limit alcohol intake, follow a low-salt and low-fat diet, control body weight, exercise regularly, and improve lifestyle. The principles of basic treatment were kept consistent between the two groups to minimize the influence of non-antithrombotic treatment factors on the study results.

#### 2.6.2 Treatment regimen in the control group

On the basis of basic treatment, the control group received clopidogrel monotherapy as antiplatelet treatment. Clopidogrel (Hangzhou Sanofi Pharmaceutical Co., Ltd., NMPA Approval No. H20056410) was administered orally at 75 mg each time, once daily. For patients who had not previously taken clopidogrel regularly and who clinically required rapid achievement of an antiplatelet effect, a loading dose could be given according to the patient's condition and the physician's judgment, followed by maintenance treatment at 75 mg/d. During treatment, medication compliance, ischemic symptoms, and bleeding risk were regularly assessed. If obvious bleeding, serious adverse reactions, or other clinical contraindications occurred, the treatment regimen was adjusted according to the patient's condition.

#### 2.6.3 Treatment regimen in the observation group

The observation group received rivaroxaban in addition to clopidogrel treatment given to the control group. The usage of clopidogrel was the same as that in the control group, namely 75 mg each time, once daily. Rivaroxaban (CSPC Ouyi Pharmaceutical Co., Ltd., NMPA Approval No. H20203077) was administered orally at a low-dose regimen of 2.5 mg each time, twice daily. Before rivaroxaban administration, renal and hepatic function, hemoglobin level, platelet count, previous bleeding history, and concomitant medications were evaluated. Rivaroxaban was not combined in patients with markedly reduced estimated glomerular filtration rate, active bleeding, severe liver disease with coagulation abnormalities, or extremely high bleeding risk. During treatment, routine blood tests and liver and kidney function were regularly rechecked, and mucocutaneous bleeding, gastrointestinal bleeding, urinary tract bleeding, and other bleeding conditions were assessed.

#### 2.6.4 Medication compliance, safety management, and follow-up

Before discharge, all patients received antithrombotic treatment education from the responsible physician or specialist nurse, including drug name, dosage, medication time, management of missed doses, common adverse reactions, warning symptoms of bleeding, and requirements for regular follow-up visits. After discharge, patients were followed up for 12 months. The follow-up methods included outpatient review, telephone follow-up, and review of rehospitalization records. The follow-up contents mainly included the use of antithrombotic drugs, medication compliance, changes in ischemic symptoms such as chest pain or chest tightness, rehospitalization, thrombotic-related adverse cardiovascular events, bleeding events, and other adverse reactions. If patients did not take medication regularly as prescribed, discontinued medication for a prolonged period, or changed the antithrombotic regimen by themselves, they were considered to have poor compliance, and whether they were included in the final efficacy analysis was determined according to the specific situation. For patients who developed minor bleeding such as gingival bleeding, epistaxis, or subcutaneous ecchymosis during follow-up, symptomatic treatment was given according to the condition, and observation was continued. If clinically relevant non-major bleeding or severe bleeding occurred, cardiovascular specialists comprehensively evaluated whether antithrombotic therapy should be suspended, adjusted, or discontinued. For patients who reported worsening chest pain, suspected myocardial infarction, stroke, or obvious bleeding symptoms during follow-up, their outpatient and emergency records, hospitalization records, laboratory test results, and imaging data were further reviewed to ensure the accuracy of endpoint event determination.

### 2.7 Observation indicators and detection methods

#### 2.7.1 Detection of thrombotic risk-related indicators

Fasting venous blood samples were collected in the morning at baseline and 6 months post-treatment. Blood was collected from the antecubital vein using a disposable vacuum blood collection system. Among the samples, 2.7 mL of blood was collected into a 3.2% sodium citrate anticoagulant tube, with an anticoagulant-to-blood ratio of 1:9. After blood collection, the tube was immediately and gently inverted 8 times for mixing, while vigorous shaking was avoided. Blood samples were sent to the laboratory department of our hospital for processing within 30 min after collection. The specimens were centrifuged at 3,000 r/min for 10 min, and the upper plasma layer was separated. D-dimer (D-D) was detected by latex-enhanced immunoturbidimetry using an automatic coagulation analyzer. Thrombin-antithrombin complex (TAT) and von Willebrand factor (vWF) were detected by enzyme-linked immunosorbent assay (ELISA). All procedures were strictly performed according to the kit instructions and the standard operating procedures of the laboratory department.

#### 2.7.2 Detection of platelet activation and thromboelastography indicators

Fasting venous blood samples were collected in the morning at baseline and 6 months post-treatment. For platelet aggregation function testing, 2.7 mL of blood was collected into a 3.2% sodium citrate anticoagulant tube. After blood collection, the tube was gently inverted for mixing, and the test was completed within 2 h after blood collection. Anticoagulated blood was first centrifuged at low speed to prepare platelet-rich plasma, followed by further centrifugation to prepare platelet-poor plasma as a reference. A platelet aggregation function analyzer was used, with adenosine diphosphate (ADP) as the inducer, to detect ADP-induced platelet aggregation rate (ADP-PAg). During the testing process, room temperature, specimen storage time, and inducer concentration were strictly controlled, and all samples were tested according to the same detection procedure. For P-selectin (CD62P) detection, another 2 mL of sodium citrate-anticoagulated venous blood was collected, gently mixed after collection, and tested within 2 h. Flow cytometry was used to detect CD62P expression on the platelet surface. An appropriate amount of whole blood sample was added with fluorescent-labeled antibodies, incubated in the dark, fixed, and then detected using a flow cytometer. During detection, gating analysis was performed using platelet-specific markers, and CD62P-positive expression levels were recorded. Negative controls and isotype controls were set for all samples to ensure the reliability of the detection results. Thromboelastography was also performed at baseline and 6 months post-treatment. A total of 2.7 mL of venous blood was collected into a 3.2% sodium citrate anticoagulant tube. After blood collection, the tube was gently inverted for mixing and allowed to stand at room temperature for 30 min before testing, and all specimens were tested within 2 h after blood collection. According to the operating instructions of the thromboelastography analyzer, sodium citrate-anticoagulated whole blood was added to the detection cup, and the corresponding activator and calcium chloride were added for recalcification before starting the test. The maximum amplitude (MA value) and reaction time (R value) were recorded. The instrument was calibrated and quality-controlled daily before testing, and sample testing was performed only after the quality control results were qualified.

#### 2.7.3 Detection of myocardial injury and cardiac functional load indicators

Fasting venous blood samples of 5 mL were collected in the morning at baseline and 6 months post-treatment and placed in procoagulant separation gel tubes. After blood collection, the samples were left standing at room temperature for 30 min. After complete coagulation, the samples were centrifuged at 3,000 r/min for 10 min to separate the serum. The separated serum was immediately used for detection. High-sensitivity cardiac troponin T (hs-cTnT), creatine kinase-MB (CK-MB), and N-terminal pro-B-type natriuretic peptide (NT-proBNP) were detected by chemiluminescence immunoassay using an automatic immunoassay analyzer. Heart-type fatty acid-binding protein (H-FABP) was detected by ELISA. All tests were performed by professional personnel in the laboratory department of our hospital according to the instructions of the instruments and reagents. Instrument calibration and internal quality control were performed before testing, and clinical samples were tested only after the quality control results met the requirements.

#### 2.7.4 Recording of thrombotic-related adverse cardiovascular events

All patients were followed up for 12 months from the date of discharge, and thrombotic-related adverse cardiovascular events were recorded. Thrombotic-related adverse cardiovascular events included nonfatal myocardial infarction, rehospitalization due to unstable angina or recurrent myocardial ischemia, ischemic stroke, coronary thrombosis, repeat revascularization, and cardiac death. If the same patient experienced multiple events during follow-up, the first event was regarded as the primary endpoint event, while each individual event was also recorded. Endpoint event data were mainly obtained through outpatient follow-up records, telephone follow-up, rehospitalization records, emergency records, and queries in the hospital information system. For patients with suspected myocardial infarction, ischemic stroke, coronary thrombosis, or cardiac death, two researchers made determinations based on electrocardiography, myocardial enzyme tests, imaging examinations, coronary angiography findings, discharge diagnosis, death certificates, and other data. If the determination results were inconsistent, a third cardiovascular specialist reviewed and made the final decision.

#### 2.7.5 Recording of bleeding events

Bleeding events in both groups were recorded during follow-up. Bleeding events included gingival bleeding, epistaxis, subcutaneous ecchymosis, gastrointestinal bleeding, urinary tract bleeding, intracranial bleeding, and bleeding at other sites. Data on bleeding events were collected through outpatient follow-up, telephone follow-up, rehospitalization records, emergency records, routine blood test results, and related imaging data. According to bleeding severity, bleeding events were classified as minor bleeding, clinically relevant non-major bleeding, and major bleeding. Major bleeding was defined as fatal bleeding, bleeding in important sites or organs, a marked decrease in hemoglobin, bleeding requiring red blood cell transfusion, or bleeding requiring surgical/interventional treatment. Clinically relevant non-major bleeding was defined as bleeding that did not meet the criteria for major bleeding but required medical attention, temporary discontinuation of medication, or adjustment of the antithrombotic treatment regimen. Minor bleeding was defined as bleeding that did not require special treatment or could be relieved with simple symptomatic treatment. The occurrence time, bleeding site, treatment method, and outcome of all bleeding events were recorded.

### 2.8 Quality control

To improve the accuracy and reliability of the study data, the following quality control measures were adopted in this study: (1) unified screening criteria for study subjects, data extraction forms, and follow-up record forms were established; (2) all coronary angiography data were independently interpreted by two cardiovascular interventional physicians; (3) all laboratory indicators were tested by the laboratory department of our hospital, with unified quality control standards implemented; (4) data entry was independently completed by two researchers, followed by cross-checking after entry; (5) follow-up endpoint events were determined by two researchers based on the original data, and disagreements were reviewed by a third researcher; (6) missing data, abnormal values, and logically inconsistent data were checked item by item, and cases that could not be confirmed were not included in the relevant analysis.

### 2.9 Statistical methods

GraphPad Prism 8 was used for data visualization and statistical analysis. Measurement data were tested for normality using the Shapiro–Wilk test and were found to conform to a normal distribution; they were expressed as mean ± standard deviation, and comparisons between groups were performed using independent-samples t-test. Within-group comparisons before and after treatment were performed using paired t-test. Count data were expressed as the number of cases or percentages, and comparisons between groups were performed using *χ*^2^ test or Fisher's exact test. Propensity score matching was used to balance baseline differences between the two groups. Major covariates were compared before and after matching, and standardized differences were used to evaluate the matching effect. A value of *P* < 0.05 was considered statistically significant.

## 3 Results

### 3.1 Comparison of general data between the two groups before and after propensity score matching

Before matching, significant differences were observed between the two groups in age and the prevalence of diabetes mellitus, suggesting baseline imbalance. After applying 1:1 nearest-neighbor propensity score matching, 70 matched pairs were generated. Baseline characteristics were well balanced between the two matched groups, with no statistically significant differences in demographic characteristics, cardiovascular risk factors, laboratory indices, angiographic findings, or concomitant medications (all *P* > 0.05), indicating satisfactory comparability after matching (Table 1).

**Table 1:** Comparison of general data between the two groups before and after propensity score matching.

Variable	Control group before matching (*n* = 82)	Observation group before matching (*n* = 76)	*t*/*x*^2^	*P*
Male	59 (71.95)	55 (72.37)	0.003	0.953
Age (years)	60.84 ± 9.42	63.72 ± 8.81	1.981	0.049
BMI (kg/m^2^)	24.58 ± 3.04	25.01 ± 3.18	0.869	0.386
Smoking history	37 (45.12)	38 (50.00)	0.376	0.540
Hypertension	43 (52.44)	51 (67.11)	3.521	0.061
Diabetes mellitus	20 (24.39)	30 (39.47)	4.149	0.042
Hyperlipidemia	36 (43.90)	43 (56.58)	2.535	0.111
Previous history of coronary heart disease	25 (30.49)	34 (44.74)	3.423	0.064
Previous history of myocardial infarction	9 (10.98)	17 (22.37)	3.724	0.054
eGFR (mL·min^−1^·1.73 m^−2^)	82.64 ± 13.82	80.15 ± 14.21	1.116	0.266
LDL-C (mmol/L)	2.71 ± 0.76	2.83 ± 0.81	0.961	0.338
LVEF (%)	59.21 ± 6.42	57.83 ± 7.06	1.287	0.200
Concomitant coronary stenosis ≥50%	34 (41.46)	43 (56.58)	3.607	0.058
PCI during hospitalization	18 (21.95)	25 (32.89)	2.385	0.123
Markis classification	–	–	5.109	0.164
Type I	10 (12.20)	17 (22.37)	–	–
Type II	16 (19.51)	20 (26.32)	–	–
Type III	34 (41.46)	23 (30.26)	–	–
Type IV	22 (26.83)	16 (21.05)	–	–
Statins	77 (93.90)	73 (96.05)	0.379	0.538
ACEI/ARB	47 (57.32)	49 (64.47)	0.847	0.357
*β*-blockers	45 (54.88)	46 (60.53)	0.515	0.473

Variable	Matched Control (*n* = 70)	Matched Observation (*n* = 70)	t/*χ*^2^	P
Male	51 (72.86)	50 (71.43)	0.036	0.85
Age (years)	62.11 ± 8.76	62.45 ± 8.51	0.238	0.812
BMI	24.76 ± 3.08	24.88 ± 3.12	0.244	0.808
Smoking history	33 (47.14)	35 (50.00)	0.115	0.735
Hypertension	39 (55.71)	44 (62.86)	0.756	0.385
Diabetes mellitus	16 (22.86)	24 (34.29)	2.104	0.147
Hyperlipidemia	32 (45.71)	38 (54.29)	1.046	0.306
Previous CHD	20 (28.57)	29 (41.43)	2.477	0.116
Previous MI	7 (10.00)	13 (18.57)	1.82	0.177
eGFR (mL·min^−1^·1.73 m^−2^)	81.52 ± 13.67	81.43 ± 13.86	0.04	0.968
LDL-C (mmol/L)	2.76 ± 0.79	2.82 ± 0.76	0.458	0.648
LVEF (%)	58.31 ± 6.65	58.02 ± 6.51	0.273	0.785
Coronary stenosis ≥50%	31 (44.29)	38 (54.29)	1.457	0.227
PCI during hospitalization	17 (24.29)	21 (30.00)	0.592	0.442
Markis classification			1.48	0.687
Type I	8 (11.43)	12 (17.14)		
Type II	14 (20.00)	17 (24.29)		
Type III	32 (45.71)	25 (35.71)		
Type IV	16 (22.86)	16 (22.86)		
Statins	65 (92.86)	67 (95.71)	0.384	0.535
ACEI/ARB	40 (57.14)	43 (61.43)	0.252	0.616
β-blockers	38 (54.29)	42 (60.00)	0.462	0.497

BMI, body mass index; eGFR, estimated glomerular filtration rate; LDL-C, low-density lipoprotein cholesterol; LVEF, left ventricular ejection fraction; PCI, percutaneous coronary intervention; ACEI/ARB, angiotensin-converting enzyme inhibitor/angiotensin II receptor blocker. The matched cohort consisted of 70 pairs generated using 1:1 nearest-neighbor propensity score matching without replacement.

### 3.2 Comparison of thrombotic risk-related indicators between the Two groups

At baseline, there were no statistically significant differences in D-D, TAT, and vWF levels between the two groups (*P* > 0.05). At 6 months post-treatment, the levels of D-D, TAT, and vWF in both groups were significantly lower than those at baseline. Further between-group comparison showed that the levels of D-D, TAT, and vWF in the observation group after treatment were lower than those in the control group, and the differences were statistically significant (*P* < 0.05). See Table 2 and Figure 3.

**Table 2:** Comparison of thrombotic risk-related indicators between the two groups before and after treatment.

Variable	Control group (*n* = 70)	Observation group (*n* = 70)	Mean difference (95% CI)	*t*	*P*
D-D (mg/L)	–	–		–	–
Baseline	0.76 ± 0.20	0.78 ± 0.21	−0.02 (−0.09 to 0.05)	0.577	0.564
6 months post-treatment	0.56 ± 0.17^a^	0.39 ± 0.13^a^	−0.17 (−0.22 to −0.12)	6.646	<0.001
TAT (μg/L)	–	–		–	–
Baseline	5.84 ± 1.72	5.91 ± 1.68	−0.07 (−0.64 to 0.50)	0.243	0.807
6 months post-treatment	4.51 ± 1.36^a^	3.42 ± 1.05^a^	−1.09 (−1.50 to −0.68)	5.307	<0.001
vWF (%)	–	–		–	–
Baseline	166.92 ± 31.86	168.35 ± 32.74	−1.43 (−12.16 to 9.30)	0.261	0.793
6 months post-treatment	145.73 ± 27.64^a^	126.48 ± 24.59^a^	−19.25 (−27.96 to −10.54)	4.353	<0.001

a indicates *P* < 0.05 compared with baseline within the same group. Mean difference represents observation group minus control group at each time point.

**Figure 3:**
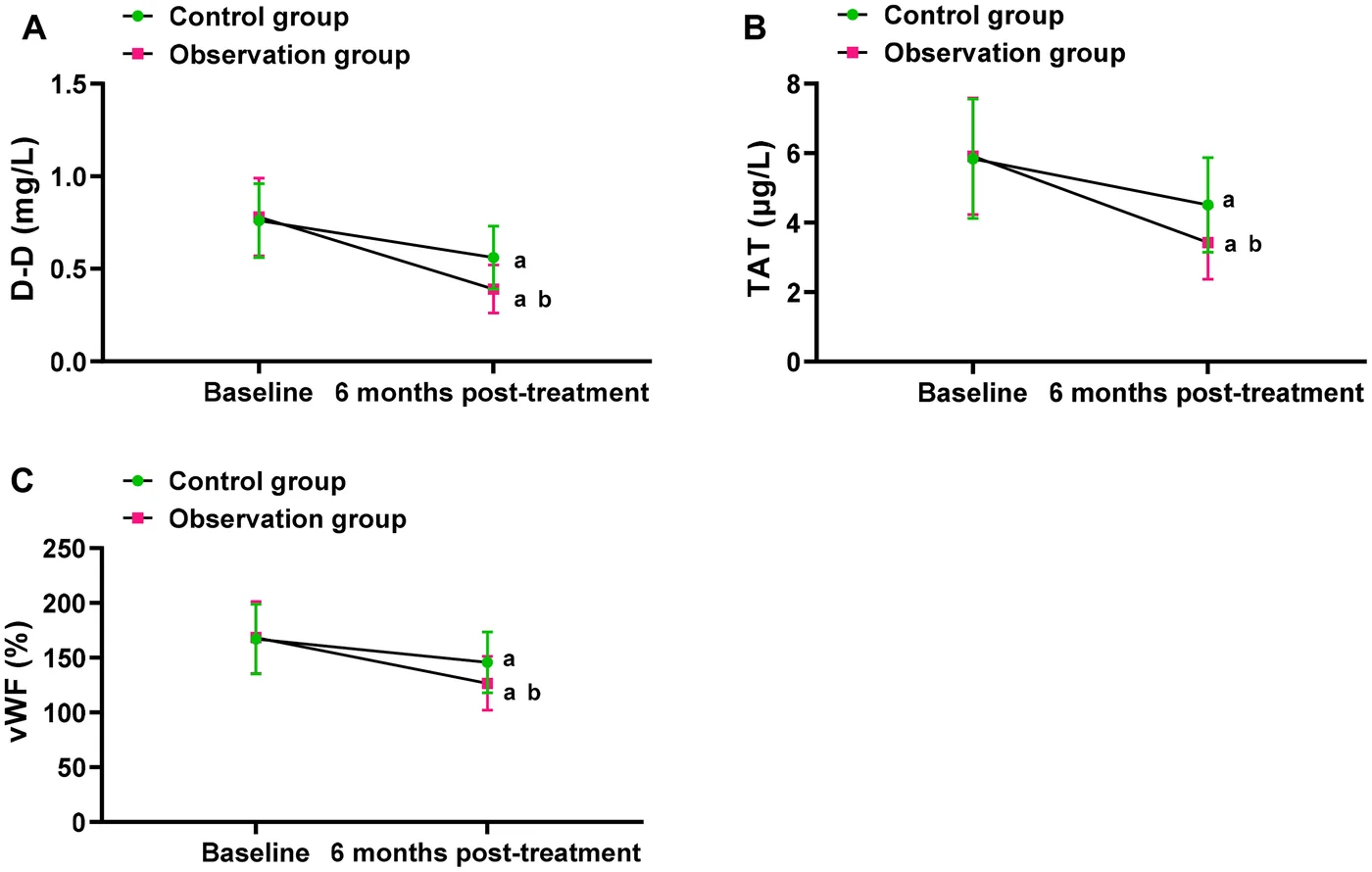
Comparison of thrombotic risk-related indicators between the two groups at baseline and 6 months post-treatment. **(A)** D-dimer (D-D**)**; **(B)** thrombin-antithrombin complex (TAT); **(C)** von Willebrand factor (vWF). Data are presented as mean ± SD. a indicates *P* < 0.05 compared with baseline within the same group; b indicates *P* < 0.05 for between-group comparison at the same time point.

### 3.3 Comparison of platelet activation and thromboelastography indicators between the Two groups

At baseline, there were no statistically significant differences in ADP-PAg, CD62P, MA value, and R value between the two groups (*P* > 0.05). At 6 months post-treatment, ADP-PAg, CD62P, and MA value decreased in both groups compared with baseline, while the R value was prolonged. Compared with the control group, the decreases in ADP-PAg [mean difference: −6.92 (95% CI: −9.69 to −4.15), *P* < 0.001], CD62P [−7.18 (95% CI: −9.79 to −4.57), *P* < 0.001], and MA value [−4.80 (95% CI: −6.47 to −3.13), *P* < 0.001] after treatment were more pronounced in the observation group, and the prolongation of the R value [+0.86 (95% CI: 0.46 to 1.26), *P* < 0.001] was more significant; the between-group differences were statistically significant. See Table 3 and Figure 4.

**Table 3:** Comparison of platelet activation and thromboelastography indicators between the two groups before and after treatment.

Variable	Control group (*n* = 70)	Observation group (*n* = 70)	Mean difference (95% CI)	*t*	*P*
ADP-PAg (%)	–	–		–	–
Baseline	58.32 ± 10.41	57.86 ± 9.95	−0.46 (−3.85 to 2.93)	0.267	0.789
6 months post-treatment	42.18 ± 8.77^a^	35.26 ± 7.83^a^	−6.92 (−9.69 to −4.15)	4.924	<0.001
CD62P (ng/mL)	–	–		–	–
Baseline	47.86 ± 9.94	48.17 ± 10.32	0.31 (−3.06 to 3.68)	0.181	0.856
6 months post-treatment	36.82 ± 8.45^a^	29.64 ± 7.18^a^	−7.18 (−9.79 to −4.57)	5.417	<0.001
MA value (mm)	–	–		–	–
Baseline	64.80 ± 5.90	65.20 ± 6.10	0.40 (−1.60 to 2.40)	0.394	0.693
6 months post-treatment	58.90 ± 5.20^a^	54.10 ± 4.80^a^	−4.80 (−6.47 to −3.13)	5.674	<0.001
R value (min)	–	–		–	–
Baseline	5.76 ± 1.11	5.71 ± 1.06	−0.05 (−0.41 to 0.31)	0.272	0.785
6 months post-treatment	6.32 ± 1.17^a^	7.18 ± 1.24^a^	0.86 (0.46 to 1.26)	4.220	<0.001

a indicates *P* < 0.05 compared with baseline within the same group. Mean difference represents observation group minus control group at each time point.

**Figure 4:**
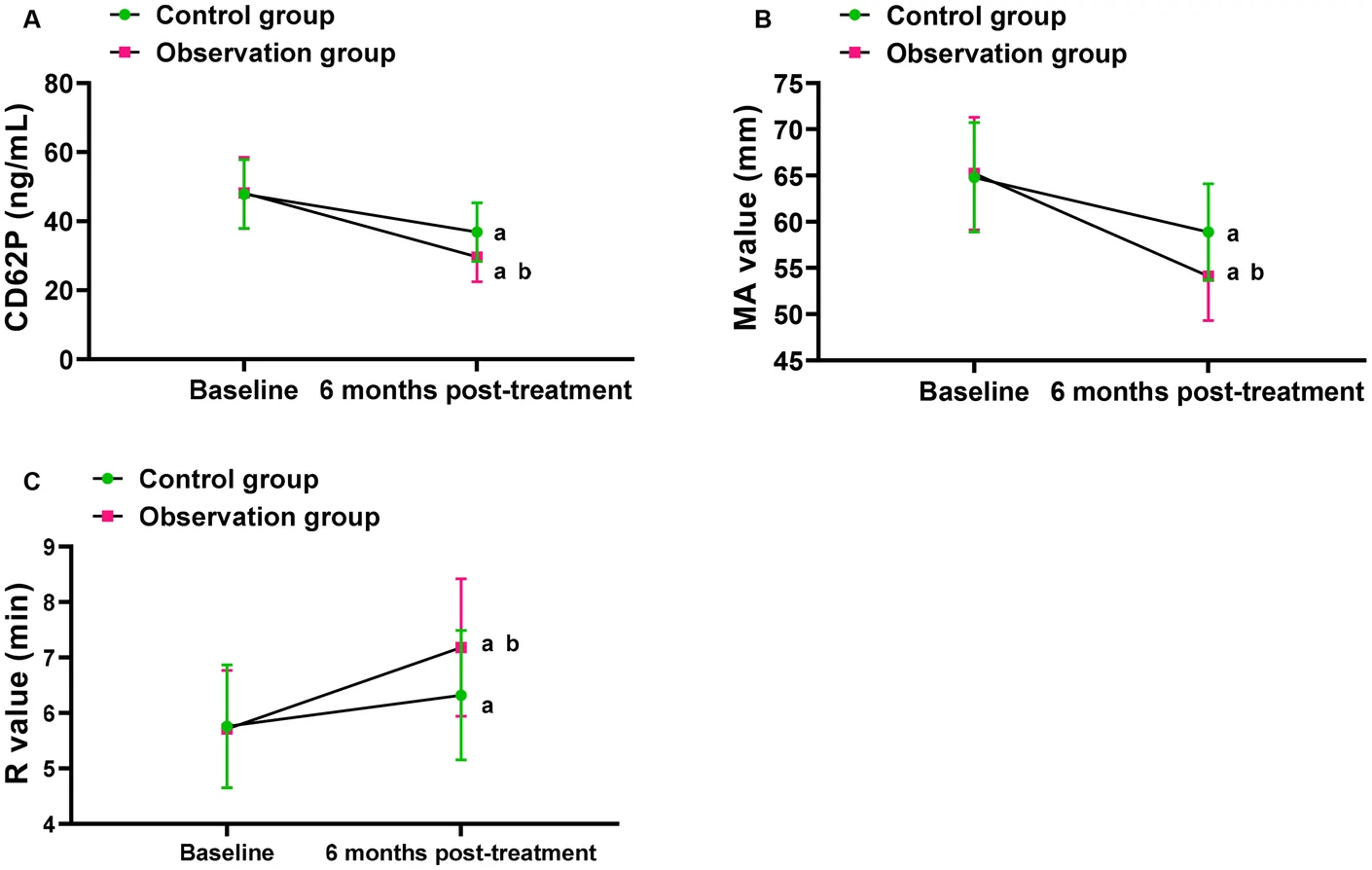
Comparison of platelet activation and thromboelastography indicators between the two groups at baseline and 6 months post-treatment. **(A)** ADP-induced platelet aggregation rate (ADP-PAg); **(B)** P-selectin (CD62P); **(C)** maximum amplitude (MA value); **(D)** reaction time (R value). Data are presented as mean ± SD. a indicates *P* < 0.05 compared with baseline within the same group; b indicates *P* < 0.05 for between-group comparison at the same time point.

### 3.4 Comparison of myocardial injury and cardiac functional load indicators between the Two groups

At baseline, there were no statistically significant differences in hs-cTnT, CK-MB, H-FABP, and NT-proBNP levels between the two groups (*P* > 0.05). At 6 months post-treatment, all the above indicators in both groups were significantly lower than those at baseline. Between-group comparison showed that the levels of hs-cTnT, CK-MB, H-FABP, and NT-proBNP in the observation group after treatment were lower than those in the control group, and the differences were statistically significant [mean differences: −5.63 (95% CI: −7.96 to −3.30), −3.46 (95% CI: −4.92 to −2.00), −1.44 (95% CI: −1.97 to −0.91), and −103.09 (95% CI: −148.06 to −58.12), respectively; all *P* < 0.001]. See Table 4 and Figure 5.

**Table 4:** Comparison of myocardial injury and cardiac functional load indicators between the two groups before and after treatment.

Variable	Control group (*n* = 70)	Observation group (*n* = 70)	Mean difference (95% CI)	*t*	*P*
hs-cTnT (ng/L)	–	–		–	–
Baseline	28.46 ± 10.12	29.11 ± 9.86	0.65 (−2.67 to 3.97)	0.384	0.700
6 months post-treatment	20.35 ± 7.84^a^	14.72 ± 5.96^a^	−5.63 (−7.96 to −3.30)	4.783	<0.001
CK-MB (U/L)	–	–		–	–
Baseline	19.64 ± 6.18	19.87 ± 6.42	0.23 (−1.87 to 2.33)	0.215	0.829
6 months post-treatment	15.32 ± 4.91^a^	11.86 ± 3.75^a^	−3.46 (−4.92 to −2.00)	4.685	<0.001
H-FABP (ng/mL)	–	–		–	–
Baseline	7.31 ± 2.44	7.45 ± 2.37	0.14 (−0.66 to 0.94)	0.344	0.731
6 months post-treatment	5.62 ± 1.81^a^	4.18 ± 1.36^a^	−1.44 (−1.97 to −0.91)	5.321	<0.001
NT-proBNP (pg/mL)	–	–		–	–
Baseline	536.58 ± 170.26	548.20 ± 165.43	11.62 (−44.12 to 67.36)	0.409	0.682
6 months post-treatment	421.73 ± 145.68^a^	318.64 ± 122.57^a^	−103.09 (−148.06 to −58.12)	4.530	<0.001

a indicates *P* < 0.05 compared with baseline within the same group. Mean difference represents observation group minus control group at each time point.

**Figure 5:**
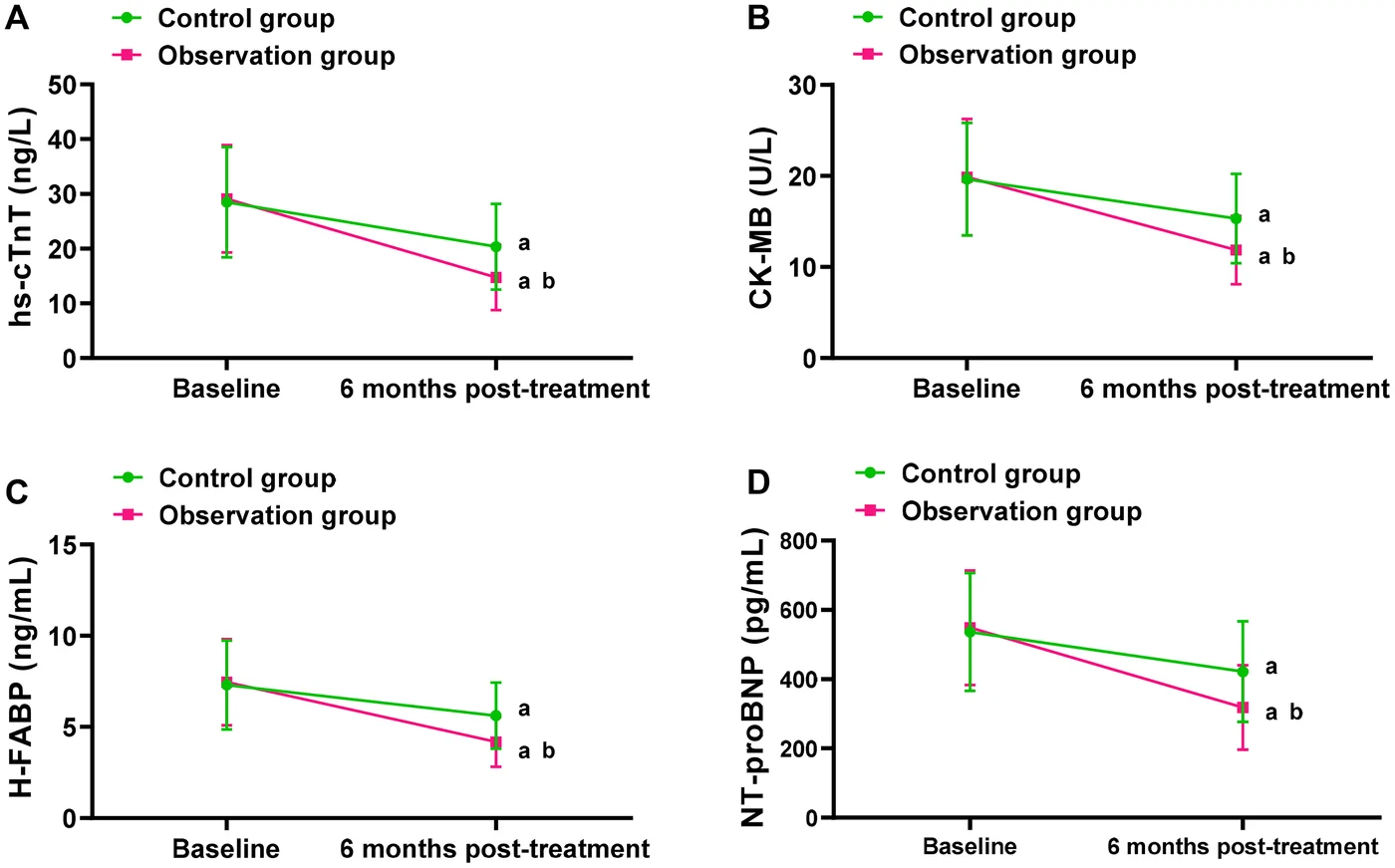
Comparison of myocardial injury and cardiac functional load indicators between the two groups at baseline and 6 months post-treatment. **(A)** High-sensitivity cardiac troponin T (hs-cTnT); **(B)** creatine kinase-MB (CK-MB); **(C)** heart-type fatty acid-binding protein (H-FABP); **(D)** N-terminal pro-B-type natriuretic peptide (NT-proBNP). Data are presented as mean ± SD. a indicates *P* < 0.05 compared with baseline within the same group; b indicates *P* < 0.05 for between-group comparison at the same time point.

### 3.5 Comparison of thrombotic-related adverse cardiovascular events between the Two groups

All patients completed 12 months of follow-up. During follow-up, 14 thrombotic-related adverse cardiovascular events occurred in the control group, with a total incidence of 20.00%; 5 thrombotic-related adverse cardiovascular events occurred in the observation group, with a total incidence of 7.14%. The total incidence of thrombotic-related adverse cardiovascular events in the observation group was significantly lower than that in the control group [risk difference: −12.86% (95% CI: −24.10% to −1.60%), *χ*^2^ = 4.912, *P* = 0.027]. In terms of individual events, the numbers of nonfatal myocardial infarction, rehospitalization due to angina or myocardial ischemia, and repeat revascularization were lower in the observation group than in the control group. For comparisons involving small cell counts (<5), Fisher's exact test was used where applicable. See Table 5.

**Table 5:** Comparison of thrombotic-related adverse cardiovascular events between the two groups during 12-month follow-up.

Event	Control group (*n* = 70)	Observation group (*n* = 70)	Risk difference (95% CI)	*P* (Fisher's exact)
Nonfatal myocardial infarction	3 (4.29)	1 (1.43)	−2.86% (−8.21% to 2.49%)	0.619
Rehospitalization due to angina or myocardial ischemia	6 (8.57)	2 (2.86)	−5.71% (−12.83% to 1.41%)	0.272
Ischemic stroke	2 (2.86)	1 (1.43)	−1.43% (−6.00% to 3.14%)	1.000
Coronary thrombosis	1 (1.43)	0 (0.00)	−1.43% (−4.22% to 1.36%)	1.000
Repeat revascularization	2 (2.86)	1 (1.43)	−1.43% (−6.00% to 3.14%)	1.000
Cardiac death	0 (0.00)	0 (0.00)	0.00%	—
Total incidence	14 (20.00)	5 (7.14)	−12.86% (−24.10% to −1.60%)	0.027 (χ^2^)

### 3.6 Comparison of bleeding events between the Two groups

During the 12-month follow-up, no intracranial bleeding, fatal bleeding, bleeding requiring surgical intervention, or massive transfusion occurred in either group. The total incidence of bleeding events was 10.00% in the control group and 17.14% in the observation group. The incidence of minor bleeding events was slightly higher in the observation group than in the control group, but the between-group difference was not statistically significant [risk difference: 7.14% (95% CI: −4.25% to 18.53%), *χ*^2^ = 1.516, *P* = 0.218]. Bleeding events in both groups were mainly gingival bleeding, epistaxis, and subcutaneous ecchymosis, and most were relieved after local treatment or short-term observation, without obvious influence on the overall treatment. For individual bleeding events with small cell counts, Fisher's exact test was applied. See Table 6.

**Table 6:** Comparison of bleeding events between the two groups during 12-month follow-up.

Event	Control group (*n* = 70)	Observation group (*n* = 70)	Risk difference (95% CI)	*P* (Fisher's exact)
Gingival bleeding	2 (2.86)	4 (5.71)	2.86% (−3.40% to 9.12%)	0.680
Epistaxis	1 (1.43)	2 (2.86)	1.43% (−3.14% to 6.00%)	1.000
Subcutaneous ecchymosis	3 (4.29)	4 (5.71)	1.43% (−5.67% to 8.53%)	1.000
Gastrointestinal bleeding	1 (1.43)	1 (1.43)	0.00% (−4.22% to 4.22%)	1.000
Urinary tract bleeding	0 (0.00)	1 (1.43)	1.43% (−1.36% to 4.22%)	1.000
Intracranial bleeding	0 (0.00)	0 (0.00)	0.00%	—
Fatal bleeding	0 (0.00)	0 (0.00)	0.00%	—
Bleeding requiring surgical intervention or massive transfusion	0 (0.00)	0 (0.00)	0.00%	—
Total incidence	7 (10.00)	12 (17.14)	7.14% (−4.25% to 18.53%)	0.218 (*χ*^2^)

## 4 Discussion

In recent years, an increasing number of studies (13–15) have suggested that CAE is not merely an imaging manifestation of vascular dilatation, but rather a complex pathological condition closely associated with atherosclerosis, endothelial injury, inflammatory response, and thrombosis. Some patients with CAE may experience recurrent chest pain, myocardial ischemia, or even acute coronary syndrome even in the absence of severe coronary artery stenosis (8), suggesting that risk assessment based solely on the degree of coronary stenosis may be insufficient. Therefore, antithrombotic treatment strategies for patients with CAE need to take into account multiple factors, including platelet activation, coagulation system activation, and microcirculatory perfusion impairment. After balancing baseline differences between the two groups using propensity score matching, this study found that rivaroxaban combined with clopidogrel reduced D-D, TAT, and vWF levels more markedly than clopidogrel monotherapy. D-D reflects fibrin formation and degradation, TAT reflects thrombin generation, and vWF is closely associated with endothelial injury and platelet adhesion (16–18). The more pronounced improvement in these indicators in the observation group suggests that combined therapy may more effectively inhibit thrombotic activity and endothelium-related thrombotic responses in patients with CAE. Previous studies on CAE have mostly emphasized the roles of slow blood flow and blood stasis within dilated vessels in thrombosis, and some studies have also indicated that patients with CAE may have higher levels of coagulation activation and inflammatory burden (19, 20). Consistent with these findings, the results of the present study further suggest that thrombotic risk in patients with CAE is not driven solely by platelet aggregation, but also involves thrombin generation and fibrin formation. Clopidogrel mainly reduces ADP-mediated platelet activation by inhibiting the P2Y12 receptor, whereas rivaroxaban reduces thrombin generation by selectively inhibiting factor Xa. Because the two drugs act on different targets, their combined use can intervene in the thrombotic process through both platelet aggregation and the coagulation cascade. This “dual-pathway antithrombotic” mechanism may be an important reason for the more pronounced improvement in thrombotic risk indicators in the observation group. Although whole-blood thrombin generation assays were not performed in this study, TAT serves as a well-established endogenous marker of thrombin generation *in vivo*. The more pronounced reduction in TAT levels observed in the observation group suggests that the addition of rivaroxaban effectively suppressed thrombin generation, which is consistent with the mechanism of factor Xa inhibition. This finding provides indirect evidence that the combined regimen attenuates coagulation cascade activation beyond the antiplatelet effect of clopidogrel alone.

The results of platelet activation and thromboelastography also support the above interpretation. This study showed that at 6 months post-treatment, ADP-PAg, CD62P, and MA value decreased in both groups compared with baseline, while the R value was prolonged, with greater improvements observed in the observation group. ADP-PAg and CD62P reflect the antiplatelet treatment effect from the perspectives of platelet aggregation function and platelet surface activation status, respectively, while MA value and R value reflect clot strength and coagulation initiation status from the perspective of the whole-blood coagulation process (21–23). The more significant decreases in ADP-PAg and CD62P in the observation group indicate that the addition of rivaroxaban did not weaken the antiplatelet effect of clopidogrel; instead, it may further reduce platelet activation by decreasing thrombin generation and thereby reducing secondary platelet activation induced by thrombin. Thrombin is not only a key enzyme in fibrin formation, but also a potent platelet agonist that can promote platelet activation and aggregation through protease-activated receptors (24). Therefore, on the basis of clopidogrel-mediated inhibition of the ADP pathway, rivaroxaban reduces thrombin generation and helps attenuate the positive feedback loop of “thrombin-platelet activation-thrombosis.” Previous studies related to stable coronary heart disease and peripheral artery disease (25, 26) have suggested that low-dose factor Xa inhibitors combined with antiplatelet therapy can reduce the risk of atherosclerotic thrombotic events. The present study applied this concept to patients with CAE, and the results showed more pronounced improvements in both platelet activation and thromboelastography parameters, suggesting that this combined strategy may be more consistent with the pathological characteristics of CAE patients, in whom slow blood flow and a hypercoagulable state coexist.

In terms of myocardial injury and cardiac functional load indicators, this study found that the levels of hs-cTnT, CK-MB, H-FABP, and NT-proBNP in the observation group after treatment were lower than those in the control group, suggesting that rivaroxaban combined with clopidogrel may have certain advantages in alleviating myocardial injury and improving cardiac load. The mechanism of myocardial injury in patients with CAE is relatively complex. In addition to insufficient blood supply caused by concomitant coronary artery stenosis, slow blood flow within dilated segments, thrombosis, distal microembolization, and microcirculatory perfusion impairment may all lead to recurrent or persistent subclinical myocardial ischemia (27). hs-cTnT and CK-MB are commonly used indicators for evaluating myocardial cell injury, H-FABP is relatively sensitive to early myocardial ischemic injury, and NT-proBNP is associated with ventricular wall tension and cardiac functional load (28, 29). In this study, the more pronounced decreases in the above indicators in the observation group may be related to the reduction of microthrombus formation within dilated coronary segments, improvement of distal microcirculatory perfusion, and attenuation of recurrent ischemic stimulation by combined antithrombotic therapy. Consistent with previous studies (30) suggesting that patients with CAE may develop myocardial ischemia due to slow blood flow and microthrombus formation, the present study further indicates, from the perspective of myocardial injury biomarkers, that the benefits of intensified antithrombotic therapy may not only be reflected in reducing macroscopic thrombotic events, but also in alleviating persistent or recurrent microcirculatory ischemic injury.

From the perspective of clinical endpoints, the 12-month follow-up results of this study showed that the total incidence of thrombotic-related adverse cardiovascular events in the observation group was lower than that in the control group, mainly reflected by reductions in nonfatal myocardial infarction, rehospitalization due to angina or myocardial ischemia, and repeat revascularization. Although the sample size of this study was limited and the incidence of some individual events was relatively low, which was insufficient for robust statistical inference for each endpoint, the overall trend of event occurrence was consistent with the improvement in laboratory indicators, suggesting that the combined antithrombotic regimen may have favorable clinical translational significance. The long-term prognosis of patients with CAE is affected by multiple factors, including thrombosis, hemodynamic abnormalities, and concomitant coronary atherosclerotic lesions. Relying solely on antiplatelet therapy may not completely reduce the risk of ischemic events. The results of this study suggest that, for patients with CAE who are suitable for intensified antithrombotic therapy after bleeding risk assessment, low-dose rivaroxaban combined with clopidogrel may help reduce the risk of thrombotic-related clinical events. Previous studies in patients with atherosclerotic cardiovascular disease (31) have shown that antiplatelet therapy combined with low-dose anticoagulation can reduce composite cardiovascular events. The results of the present study are consistent with this overall direction, but the study population focused on the special subgroup of patients with CAE, thereby providing certain supplementary value (32).

The safety of antithrombotic therapy is always a key concern in clinical application. While rivaroxaban combined with clopidogrel enhances the antithrombotic effect, it may theoretically increase the risk of bleeding. The results of this study showed that the total incidence of bleeding events in the observation group was slightly higher than that in the control group, but the difference between the two groups was not statistically significant. Moreover, no intracranial bleeding, fatal bleeding, bleeding requiring surgical intervention, or massive transfusion occurred in either group. Bleeding events were mainly minor bleeding events such as gingival bleeding, epistaxis, and subcutaneous ecchymosis, which were relieved after symptomatic treatment or short-term observation. These results suggest that, under the premise of strict patient screening, exclusion of high-bleeding-risk populations, and standardized follow-up management, rivaroxaban combined with clopidogrel has acceptable safety in patients with CAE. It should be noted that the combined treatment regimen in this study used low-dose rivaroxaban, which may be an important reason for avoiding a significant increase in severe bleeding while enhancing the antithrombotic effect. It should be emphasized that patients with coagulation disorders, including haemophilia, were explicitly excluded from this study, and such a combined antithrombotic regimen would be absolutely contraindicated in these populations due to the prohibitively high bleeding risk. In clinical practice, for patients with CAE who are considered for combined antithrombotic therapy, factors such as age, renal function, previous bleeding history, gastrointestinal disease, concomitant medications, and compliance should be fully evaluated, and bleeding risk should be dynamically monitored during treatment to achieve a balance between ischemic benefit and bleeding risk.

## 5 Clinical significance and limitations

From the perspective of clinical outcomes, the 12-month follow-up results showed that the incidence of thrombotic-related adverse cardiovascular events was lower in the rivaroxaban plus clopidogrel group than in the clopidogrel monotherapy group, mainly reflected by reductions in nonfatal myocardial infarction, rehospitalization for angina or myocardial ischemia, and repeat revascularization. Although the limited sample size and relatively low event rate prevented robust analysis of individual endpoints, the overall trend was consistent with the improvements observed in thrombotic biomarkers, platelet activation parameters, and myocardial injury indicators.

CAE is characterized by abnormal coronary dilatation, impaired blood flow dynamics, and a potential prothrombotic state. Therefore, antiplatelet therapy alone may not completely address the multiple mechanisms involved in thrombus formation. By simultaneously inhibiting platelet activation and factor Xa-mediated thrombin generation, rivaroxaban combined with clopidogrel may provide broader inhibition of thrombotic pathways. However, given the retrospective design and limited sample size, these findings should be interpreted as exploratory and require confirmation in prospective studies.

The safety of intensified antithrombotic therapy remains an important consideration. In this study, although bleeding events were numerically higher in the combination therapy group, the difference was not statistically significant, and no intracranial bleeding, fatal bleeding, or major bleeding requiring intervention occurred. These findings suggest that low-dose rivaroxaban combined with clopidogrel may have an acceptable safety profile in carefully selected patients with CAE. Nevertheless, individualized assessment of ischemic and bleeding risks remains essential before initiating combined antithrombotic therapy.

This study has several limitations. First, this was a single-center retrospective cohort study, and residual confounding and selection bias cannot be completely excluded despite the use of propensity score matching. Potential unmeasured factors, including coronary slow-flow severity, hemodynamic characteristics of ectatic segments, plaque features, lifestyle modifications, and medication adherence, may have influenced the outcomes. Second, the relatively small sample size and low number of clinical events limited the statistical power for individual endpoint analyses. Therefore, the findings should be considered hypothesis-generating rather than definitive evidence of treatment efficacy. Third, the follow-up duration was limited to 12 months, and longer-term benefits and bleeding risks associated with combined antithrombotic therapy remain unclear. In addition, laboratory biomarkers were assessed only at baseline and 6 months, limiting evaluation of the temporal changes after treatment initiation. Fourth, subgroup analyses according to CAE extent, Markis classification, coronary slow flow, and concomitant coronary stenosis were not performed. Future multicenter prospective studies with larger sample sizes and longer follow-up are needed to identify patients who may derive the greatest benefit from combined therapy and to determine the optimal treatment duration and risk-benefit balance.

## 6 Conclusion

In patients with CAE, rivaroxaban combined with clopidogrel was associated with more favorable improvements in thrombotic risk indicators, platelet activation markers, thromboelastography parameters, and myocardial injury markers compared with clopidogrel monotherapy at 6-month follow-up. The 12-month follow-up results further suggested that this combined regimen reduced the incidence of thrombotic-related adverse cardiovascular events, without a statistically significant increase in overall bleeding events, and no major or fatal bleeding events were observed in either group. These findings suggest that the dual-pathway inhibition strategy may better address the complex thrombotic pathophysiology of CAE and may serve as a potential therapeutic option for patients with CAE at elevated thrombotic risk. However, given the single-center, retrospective design and limited sample size, these findings are exploratory in nature and require confirmation through multicenter, large-sample, prospective randomized controlled trials with extended follow-up duration to establish the long-term efficacy, safety, and optimal treatment duration of this combined regimen.

## Data Availability

The original contributions presented in the study are included in the article/Supplementary Material, further inquiries can be directed to the corresponding author.
